# Associations between cardiovascular diseases and cancer mortality: insights from a retrospective cohort analysis of NHANES data

**DOI:** 10.1186/s12889-024-18498-7

**Published:** 2024-04-15

**Authors:** Chenliang Ge, Zhiyuan Jiang, Binghua Long, Qingjian Lu, Yan He

**Affiliations:** Department of Cardiology, The First Affiliated Hospital of Guangxi Medical University, Nanning, Guangxi Zhuang Autonomous Region China

**Keywords:** Cardiovascular disease, Cancer mortality, National health and nutrition examination survey, Heart failure

## Abstract

**Background:**

This study explored the association of cardiovascular disease (CVD) with cancer mortality risk in individuals with or without a history of cancer, to better understand the interplay between CVD and cancer outcomes.

**Methods:**

Utilizing data from the National Health and Nutrition Examination Survey (NHANES) spanning 1999 to 2018, a retrospective cohort analysis was conducted. This analysis accounted for the survey’s complex design to ensure national representativeness. The association of CVD with cancer mortality was assessed through multivariable Cox proportional hazards models.

**Results:**

The present study included 59,653 participants, of whom 54,095 did not have cancer and 5558 had a history of cancer. In individuals without cancer, heart failure (HF) was associated with an increased risk of mortality from cancer (HR, 1.36; 95% CI, 1.09–1.69; *P* = 0.005). In participants with cancer, HF correlated with a higher risk of mortality from cancer (HR, 1.76; 95% CI, 1.32–2.34; *P* < 0.001). Diabetes (DM), hypertension (HBP) and coronary heart disease (CHD) were not significantly associated with an increased risk of mortality from cancer. Significant differences were observed in the interaction between cancer and CHD (HR, 0.68; 95% CI, 0.53–0.87; *P* = 0.002). For cancer and HBP, a similar trend was noted (HR, 0.75; 95% CI, 0.62–0.91; *P* = 0.003). No significant differences were found in interactions between HF, DM and cancer.

**Conclusions:**

HF was associated with an increased risk of mortality from cancer, regardless of cancer history, while HBP, CHD and DM showed no significant association. These findings underscore the importance of understanding the mechanisms behind the increased risk of cancer mortality following HF.

**Supplementary Information:**

The online version contains supplementary material available at 10.1186/s12889-024-18498-7.

## Background

Cancer and cardiovascular disease (CVD) are the leading non-communicable causes of global morbidity and mortality. In 2015, CVD resulted in 17.7 million deaths globally, while cancer was responsible for 8.8 million deaths [1–3]. Since the 1990s, there has been a notable decline in cancer-related mortality, with projections indicating that the number of cancer survivors in the United States will exceed 26 million by 2040 [4–7]. This growing population of cancer survivors faces an increased risk of developing CVD, with cardiac risk factors significantly influencing treatment-related cardiotoxicity. Both CVD and cancer share common risk factors, such as obesity and diabetes (DM), suggesting a potential shared pathobiology—a concept supported by emerging evidence [8, 9]. This intersection of cancer and CVD has led to the development of the specialized field of cardio-oncology [10–12].

Despite the recognized link between cancer and CVD, the evidence guiding clinical decisions in cardio-oncology remains sparse. Extensive research has been conducted on cancer treatment-induced cardiotoxicity, the impact of pre-existing CVD on cancer mortality, especially among cancer patients, is less understood [13–15]. Recognizing this gap, our study seeks to provide empirical evidence on the role of CVD in cancer mortality, utilizing data from the National Health and Nutrition Examination Survey (NHANES). We hypothesize that CVD significantly increases the risk of cancer mortality and aimed to exam the association between CVD and cancer mortality in individuals with or without a history of cancer.

## Methods

### Study population

**Fig. 1:**
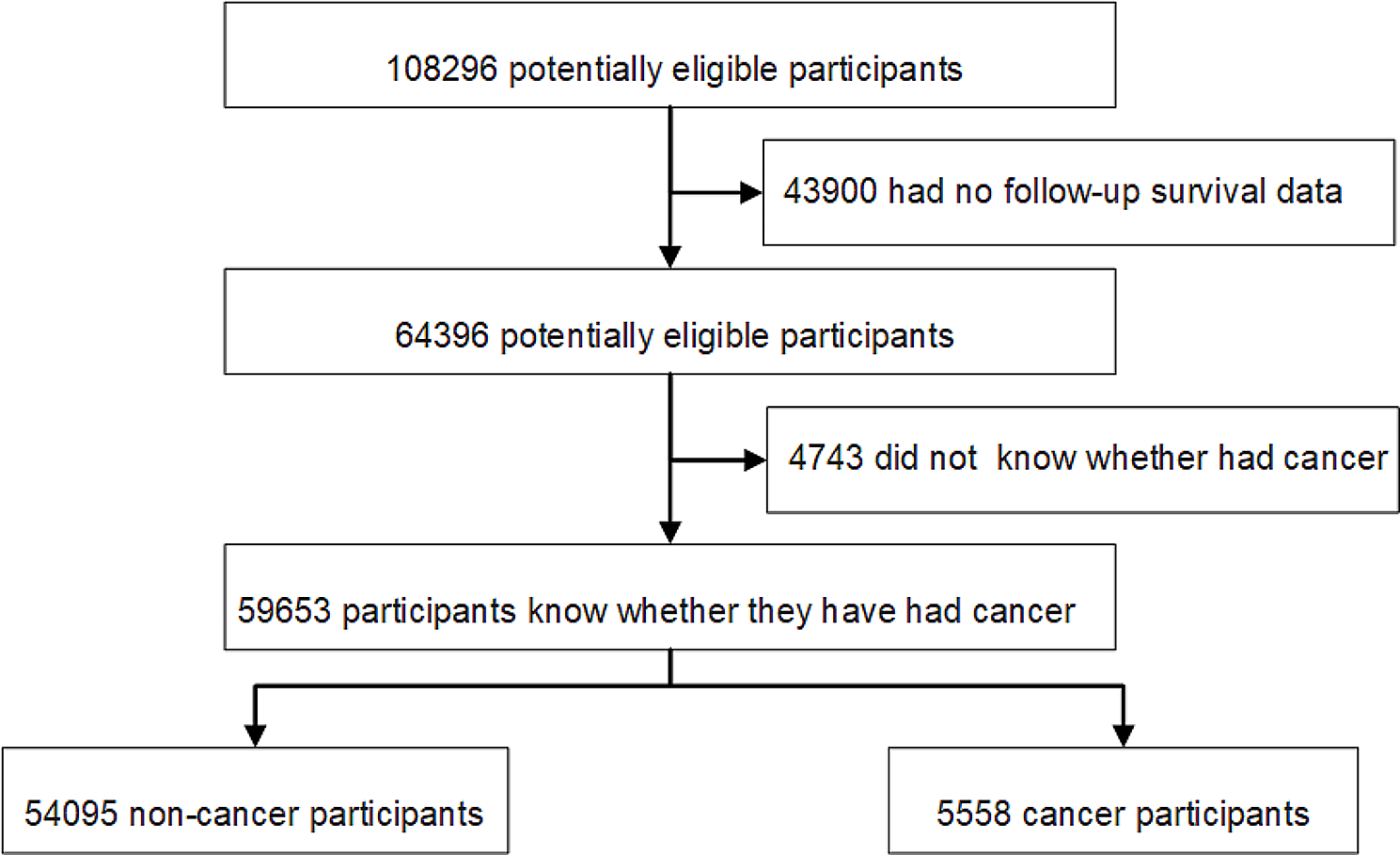
Flow diagram of study sample selection

This study utilized data from the NHANES, a representative, multistage, and stratified health survey conducted in the United States [16–20]. This study received ethical approval from the National Center for Health Statistics (NCHS) Institutional Review Board and informed consent was obtained from all participants. The research adhered to the Tenets of the Declaration of Helsinki. Ethical considerations have been rigorously followed to ensure that participants confidentiality was not impacted. We included participants from the NHANES database spanning 1999 to 2018, exclusion criteria were set to omit individuals without clear cancer status or those missing follow-up survival data. Participants were categorized into cancer and non-cancer groups based on physician-reported cancer diagnoses (Fig. 1). NCHS linked the survey data with death certificate records from the National Death Index (NDI) for mortality follow-up. Follow-up time was calculated in person-months from the interview date to either the date of death, the end of the mortality follow-up period, or December 31, 2019, whichever occurred first. The linked mortality files classified causes of death into nine categories using (ICD)-10 codes. Our primary focus was on deaths due to malignant neoplasms (ICD-10 codes: C00-C97) and all-cause mortality.

### Sociodemographic characteristics and covariates

Participants provided information on age, gender, race and ethnic group (Mexican American, Other Hispanic, Non-Hispanic White, Non-Hispanic Black, Other Race), education level (< High school, High school, some college or Associates degree, College graduate) and marital status (never married, married or living with a partner, separated or divorced or widowed). The ratio of family income to the poverty level was categorized as < 1, 1 to 3, or > 3. Smoking status was categorized as current, past or never. Body mass index (BMI), calculated as the weight in kilograms divided by the square of the height in meters, was classified into three weight-status groups: normal (BMI < 25), overweight (BMI 25 ∼ 30), or obese (BMI ≥ 30). Creatinine data were obtained from the original database. The presence of various comorbidities, such as DM, hypertension (HBP), coronary heart disease (CHD), heart failure (HF), stroke, chronic bronchitis and chronic liver disease, was determined based on reported diagnoses from a physician.

### Statistical analysis

We employed complex survey design adjustments from NHANES data to ensure representative estimates for the US population, accounting for sample weights, clustering, and stratification [21, 22]. Data analysis was conducted using R software version 4.3.1. Categorical variables were analyzed using Rao-Scott adjusted Chi-square test and continuous variables were analyzed using weighted mean comparisons. Kaplan-Meier survival curves provided weighted comparisons of the cumulative incidence of cancer-related deaths and all-cause deaths. We rigorously tested the proportional hazards assumption through the examination of martingale residuals and the application of time-dependent covariate tests. Cox proportional hazards models, incorporating survey sample weights, were utilized to estimate hazard ratio (HR) for cancer mortality, adjusting for potential confounders including gender, age, BMI, race, education, marital status, income level and CVD conditions including CHD, HF, HBP, DM. Missing data were addressed using the fully efficient fractional imputation technique, with less than 3% missing values for most variables, except for BMI (6.3% missing), family income-to-poverty ratio (9.9% missing) and Creatinine (11.8% missing) [23]. Sensitivity analyses excluded subjects with missing values in BMI, marital status, creatinine, and DM, HBP, CHD, HF statuses. Statistical significance was set at *p* ≤ 0.05.

## Results

### Participants characteristics

The study cohort comprised 59,653 individuals who were categorized into the non-cancer group (*N* = 54,095) and the cancer group (*N* = 5558). Table 1 presents the clinical characteristics of non-cancer and cancer participants. Compared to non-cancer participants, those with cancer were older, had a higher proportion of females, and exhibited elevated systolic blood pressure levels. Diastolic blood pressure levels were lower in the cancer group. A lower percentage of smokers was noted in the cancer group, and this group had a shorter follow-up time. In terms of education level, individuals with or above a college education were more prevalent in the cancer group, while those with an education level below high school were less frequent in the cancer group. The proportion of participants living alone was notably higher in the cancer group. Creatinine levels were higher in cancer participants than in non-cancer participants. Cancer participants, in comparison with non-cancer participants, were significantly more likely to have DM (15.5% vs. 8.2%, respectively; *P* < 0.001), HBP (50.4% vs. 28.3%, respectively; *P* < 0.001), HF (6.2% vs. 2.0%, respectively; *P* < 0.001), CHD (8.1% vs. 3.0%, respectively; *P* < 0.001), Stroke (6.5% vs. 2.4%, respectively; *P* < 0.001), Chronic bronchitis (11.2% vs. 5.6%, respectively; *P* < 0.001) and Liver condition (5.3% vs. 3.3%, respectively; *P* < 0.001). Among all cancer participants, skin cancer had the highest proportion, accounting for 28.3%, followed by breast cancer (15.8%), prostate cancer (9.4%), cervix cancer (8.1%), melanoma (7.4%), colon cancer (4.7%), uterus cancer (3.6%), lung cancer (2.2%), other types of cancer accounting for 20.5%.

**Table 1:** Clinical and demographic characteristics of NHANES participants by cancer status, 1999–2018^*^

Characteristic	Cancer participants		Non-cancer participants	*P* value
**N**	5558		54,095	
**Estimated N**	22,239,302		214,853,630	
**Age**	62.4(14.8)		45.2(16.4)	< 0.001
**Sex**				< 0.001
Male	42.00%		48.70%	
Female	58.00%		51.30%	
**BMI**				0.1505
< 25	30.30%		31.60%	
25 ∼ 30	34.50%		33.40%	
> 30	35.10%		35.10%	
**Pulse(/min)**	71.4(12)		72.8(12.1)	< 0.001
**SBP (mmHg)**	128.2(19.9)		122(17.6)	< 0.001
**DBP (mmHg)**	69(13.5)		71(12.3)	< 0.001
**Follow time (year)**	8.3(5.2)		10.4(5.5)	< 0.001
**Cigarette use**				< 0.001
Current	26.80%		40.50%	
Past	3.90%		8.50%	
Never	69.30%		51.00%	
**Race/Ethnicity**				< 0.001
Non-Hispanic White	86.30%		66.40%	
Other Race	13.70%		33.60%	
**Ratio of family income to poverty level**				
<1	9.90%		14.00%	
1 ∼ 3	34.90%		36.60%	
>3	55.20%		36.60%	
**Education level**				< 0.001
< High school	15.20%		17.70%	
High school/GED	22.70%		24.30%	
Some college or Associates degree	30.60%		30.80%	
College graduate	31.50%		27.00%	
**Marita Status**				< 0.001
Never married	5.60%		19.10%	
Married or living with partner	65.70%		63.30%	
Divorced, separated, or widowed	28.60%		17.50%	
**Laboratory test**				
Creatinine(mg/dL)	0.92(0.4)		0.85(0.4)	< 0.001
**Pre-exist comorbidities**				
**DM**				< 0.001
Yes	15.50%		8.20%	
No	84.50%		91.80%	
**HBP**				< 0.001
Yes	50.40%		28.30%	
No	49.60%		71.70%	
**HF**				< 0.001
Yes	6.20%		2.00%	
No	93.80%		98.00%	
**CHD**				< 0.001
Yes	8.10%		3.00%	
No	91.90%		97.00%	

*Data were shown as weighted percent or weighted mean. NHANES: National Health and Nutrition Examination Survey; SBP: systolic blood pressure; DBP: diastolic blood pressure; ALT: Alanine aminotransferase; AST: Aspartate aminotransferase; LDL: low-density lipoprotein; BMI: body-mass index; DM: diabetes; HF: heart failure; CHD: coronary heart disease; HBP: hypertension

Clinical and demographic details between participants with and without a cancer diagnosis were illustrated. The *P* values indicate the statistical significance of differences between groups, calculated using design-adjusted Rao-Scott χ2 tests for categorical variables and weighted analysis of variance for continuous variables

### Association of CVD with cancer mortality

Examination of martingale residuals showed the proportional hazards assumption is reasonable for data of the present study (Supplemental figure). Among all participants, presence of cancer was associated with higher risk of cancer mortality among cancer participants, when compared with participants without cancer (HR 2.35, 95%CI 2.14 ∼ 2.58, *P* < 0.001). Presence of HF was associated with higher risk of cancer mortality among all participants, when compared with participants without HF (HR 1.52, 95%CI 1.34 ∼ 1.71, *P* < 0.001). DM, HBP, CHD were not associated with significant increased cancer mortality risk. There was statistically significant difference in the associations of cancer× CHD interaction (HR 0.68, 95%CI 0.53 ∼ 0.87, *P* = 0.002) and cancer× HBP interaction (HR 0.75, 95%CI 0.62 ∼ 0.91, *P* = 0.003). There was no statistically significant difference in the associations of cancer× HF interaction (*P* = 0.891) and cancer× DM interaction (*P* = 0.56) (Table 2).

**Table 2:** The association between cardiovascular conditions and cancer mortality in NHANES participants

	All participants				Interaction with cancer		
Covariates	HR	95% CI	*P* value		HR	95% CI	*P* value
**Cancer**							
No	1.00 (Ref)				—	—	—
Yes	2.35	2.14 ∼ 2.58	< 0.001				
**HF**							
No	1.00 (Ref)				—	—	0.891
Yes	1.52	1.34 ∼ 1.71	< 0.001				
**Diabetes**							
No	1.00 (Ref)				—	—	0.560
Yes	0.99	0.83 ∼ 1.17	0.931				
**CHD**							
No	1.00 (Ref)				0.68	0.53 ∼ 0.87	0.002
Yes	0.94	0.83 ∼ 1.08	0.394				
**HBP**							
No	1.00 (Ref)				0.75	0.62 ∼ 0.91	0.003
Yes	1.07	0.93 ∼ 1.22	0.322				

* Data was shown as HR (95% CI). Ref, reference; HR: hazard ratio; CI, confidence interval; DM: diabetes; HF: heart failure; CHD: coronary heart disease; HBP: hypertension

Survey sample weights were taken into consideration in the Cox models accompanying the NHANES data. It examines the association of HF, DM, CHD and HBP with cancer mortality and the interaction with cancer status, adjusting for gender, age, BMI, race, education, marital status, income level and pre-exist comorbidities including cancer, CHD, HF, HBP, DM

Among non-cancer participants, presence of HF was associated with higher risk of cancer mortality among cancer participants, when compared with participants without HF (HR 1.36, 95%CI 1.09 ∼ 1.69, *P* = 0.005). DM, HBP, CHD were not associated with significant increased cancer mortality risk. Among cancer participants, presence of HF was associated with higher risk of cancer mortality among cancer participants, when compared with participants without HF (HR 1.76, 95%CI 1.32 ∼ 2.34, *P* < 0.001). DM, HBP, CHD were not associated with significant increased cancer mortality risk (Table 3).

**Table 3:** Hazard Ratios for cancer mortality associated with cardiovascular conditions in cancer and non-cancer NHANES participants*

	Cancer participants				Non-Cancer participants		
Covariates	HR	95% CI	*P* value		HR	95% CI	*P* value
**HF**							
No	1.00 (Ref)				1.00 (Ref)		
Yes	1.76	1.32 ∼ 2.34	< 0.001		1.36	1.09 ∼ 1.69	0.005
**Diabetes**							
No	1.00 (Ref)				1.00 (Ref)		
Yes	0.99	0.71 ∼ 1.38	0.973		0.99	0.81 ∼ 1.22	0.923
**CHD**							
No	1.00 (Ref)				1.00 (Ref)		
Yes	0.75	0.51 ∼ 1.11	0.147		1.07	0.89 ∼ 1.29	0.442
**HBP**							
No	1.00 (Ref)				1.00 (Ref)		
Yes	0.95	0.77 ∼ 1.17	0.621		1.12	0.98 ∼ 1.27	0.084

* Data was shown as HR (95% CI). Ref, reference; HR: hazard ratio; CI, confidence interval; DM: diabetes; HF: heart failure; CHD: coronary heart disease; HBP: hypertension

Survey sample weights were taken into consideration in the Cox models accompanying the NHANES data. It examines the association of HF, DM, CHD and HBP with cancer mortality, adjusting for gender, age, BMI, race, education, marital status, income level and pre-exist comorbidities including CHD, HF, HBP, DM

**Fig. 2:**
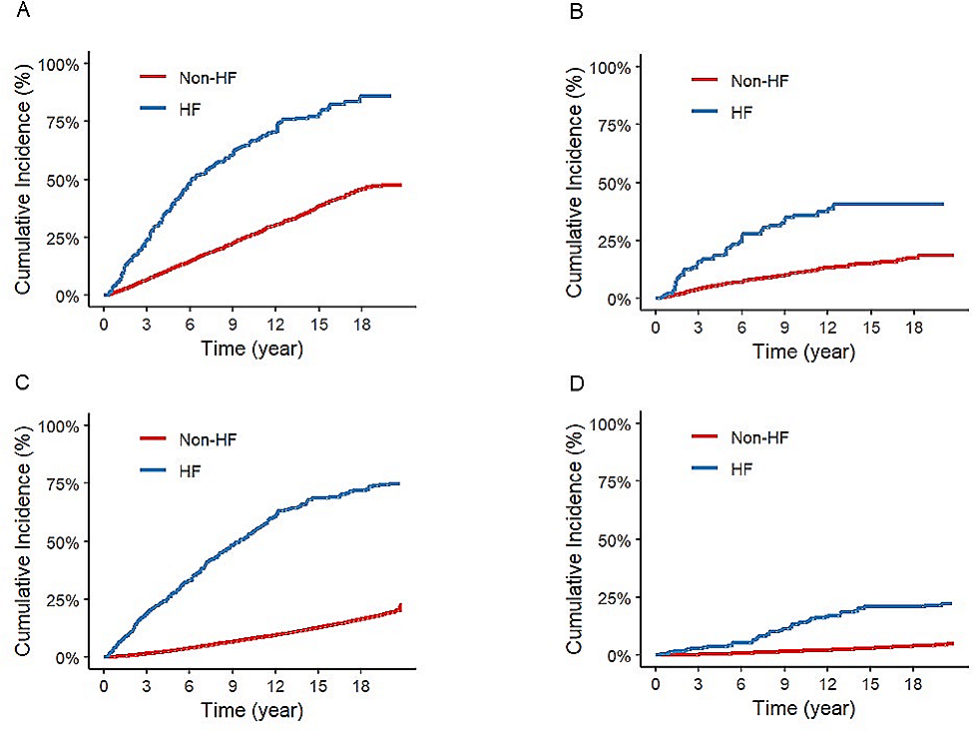
Kaplan-Meier Survival Curves for All-Cause and cancer Mortality. Cumulative mortality rates were estimated with use of imputation-adjusted survey weights. (**A**) All-cause mortality among cancer participants with heart failure (HF) versus those without. (**B**) Cancer mortality among cancer participants with HF versus those without. (**C**) All-cause mortality among non-cancer participants with HF versus those without. (**D**) Cancer mortality among non-cancer participants with HF versus those without. Mortality rates are adjusted for imputation and survey weights to reflect the NHANES cohort accurately

Kaplan-Meier curves showed cumulative all-cause mortality and cancer mortality. Cancer participants with HF had a higher all-cause mortality compared with cancer participants without HF. Cancer participants with HF also had a higher cancer mortality compared with cancer participants without HF. Non-cancer participants with HF had a higher all-cause mortality compared with non-cancer participants without HF. Non-cancer participants with HF also had a higher cancer mortality compared with non-cancer participants without HF (Fig. 2).

In sensitivity analyses, presence of HF was associated with higher risk of cancer mortality among all participants, when compared with participants without HF (HR 1.56, 95%CI 1.18 ∼ 2.06, *P* < 0.001). DM, HBP, CHD were not associated with significant increased cancer mortality risk. Among non-cancer participants, presence of HF was associated with higher risk of cancer mortality among cancer participants, when compared with participants without HF (HR 1.47, 95%CI 1.02 ∼ 2.13, *P* = 0.03). Among cancer participants, presence of HF was associated with higher risk of cancer mortality among cancer participants, when compared with participants without HF (HR 1.69, 95%CI 1.03 ∼ 2.79, *P* = 0.037).

## Discussion

The present study embarked on an exploration of the associations between CVD and cancer mortality, leveraging a comprehensive retrospective cohort analysis of data from NHANES spanning 1999 to 2018. Confirming our hypotheses, we found that HF was associated with a 37% increased risk of cancer mortality in participants without cancer and a 73% increase in those with cancer, compared to those without HF. The findings of this investigation revealed that HF is a notable predictor of increased cancer mortality risk irrespective of cancer history, a discovery that underscores the intricate and potentially bidirectional relationship between HF and cancer. Conversely, DM, HBP, and CHD did not exhibit a statistically significant association with cancer mortality, highlighting the unique position of HF within the spectrum of CVD affecting cancer outcomes.

Our findings concur with prior research that has indicated a heightened cancer risk associated with HF [24–27] and may be linked to shared risk factors, such as the association of chronic kidney disease with increased cancer risk in the elderly [28]. We observed that common risk factors like HBP, obesity, DM, and tobacco use are shared between cancer and heart failure. Similarly, Symptoms such as fatigue, dyspnea, and weight loss also present in both HF and cancer, adding complexity to their management [29–32]. Koene et al. elucidated the shared risk factors and biological mechanisms between CVD and cancer, suggesting a unified pathobiological framework that may contribute to the co-occurrence of these diseases [9]. Chronic inflammation and immune modulation in HF could promote tumor progression. Experimental models have shown a causal relationship between ischemic HF and tumor growth, possibly mediated by factors released from failing myocardium [8, 33–37]. Sympathetic nervous system activation in HF, as observed in breast cancer mouse models, is associated with increased metastasis, which can be mitigated by beta-blocker therapy [38]. This suggests a potential therapeutic role for beta-blockers in cancer patients with elevated heart rates [39].

Our study uniquely identified that CHD and HBP demonstrated an interactive effect with cancer, which may provide a protective influence. This interaction may be linked to the protective effects of medications used in the treatment of HBP and CHD. Angiotensin Receptor Blocker (ARB) hold anti-tumor potential by inhibiting the action of angiotensin II, as do Angiotensin-Converting Enzyme Inhibitors (ACEI), which block the generation of angiotensin II and are considered to have anti-tumor effects. Long-term ARB and ACEI use was significantly associated with a reduced risk of incident cancer [40]. Statins, such as atorvastatin, simvastatin, rosuvastatin and pravastatin, have demonstrated anticancer activity across various cancer types in laboratory studies. These drugs exert direct effects on cancer cells, influencing tumor initiation, progression, metastasis, and response to therapy. While the role of statins in cancer prevention is debated, robust research confirms their potential as repurposed drugs in the fight against cancer. Recent systematic reviews and meta-analyses indicate that statin treatment is linked to a decreased risk of overall mortality and cancer-specific mortality in advanced-stage cancer patients. The multifaceted effects of statins, including antiproliferative and apoptotic-inducing properties, position them as promising agents in cancer therapy, introducing innovative perspectives and novel treatment targets [41–45].

Our study, while providing valuable insights into the association between CVD and cancer mortality, is subject to several limitations. Firstly, relying on data from NHANES introduces potential biases such as recall bias and inaccuracies in self-reported health and lifestyle factors. The retrospective cohort design, though robust, cannot establish causality between CVD conditions and cancer mortality, highlighting the need for prospective or randomized controlled designs in future research. Despite adjusting for multiple confounders, residual or unmeasured confounding factors could still influence the observed associations. The study primarily focuses on HF, DM, HBP and CHD, with limited data on other CVD conditions and specific types of cancer, which constrains our understanding of these associations. Additionally, the span of data from 1999 to 2018 encompasses significant changes in healthcare and lifestyle, the implications of which may not be fully captured in our analysis. Addressing these limitations in future studies is crucial for refining our understanding of the complex interplay between cardiovascular health and cancer outcomes.

## Conclusions

In conclusion, we found that HF exhibited an elevated risk of cancer mortality, irrespective of a patient’s cancer history. This association underscores the importance of integrating cardiovascular health management into cancer care strategies. Conversely, DM, HBP and CHD did not demonstrate a significant correlation with increased cancer mortality risk, highlighting the specificity of HF ‘s impact on cancer outcomes. Our findings contribute to the burgeoning field of cardio-oncology, emphasizing the need for a multidisciplinary approach to patient care that addresses both cardiovascular health and cancer risk. The nuanced understanding of the relationship between specific cardiovascular conditions and cancer mortality could lead to more effective prevention, management, and treatment strategies that holistically address patient health. As the interplay between CVD and cancer continues to reveal its complexity, ongoing research in this intersection is imperative for advancing patient care and improving outcomes.

### Electronic supplementary material

Below is the link to the electronic supplementary material.

Supplementary Material 1

## Data Availability

The data and the simulation results that support the findings of this study are available in Figshare with the identifie. All National Health and Nutrition Examination Survey data were accessed from https://www.cdc.gov/nchs/nhanes.htm.

## Abbreviations

CVD: Cardiovascular disease
NHANES: National Health and Nutrition Examination Survey
HF: Heart failure
DM: Diabetes
HBP: Hypertension
CHD: Coronary heart disease
NCHS: National Center for Health Statistics
NDI: National Death Index
ICD: International Classification of Diseases
BMI: Body mass index
ARB: Angiotensin Receptor Blocker
ACEI: Angiotensin-Converting Enzyme Inhibitors
