# Supplementary material for: Associations between cardiovascular diseases and cancer mortality: insights from a retrospective cohort analysis of NHANES data
Source: BMC Public Health. 2024 Apr 15;24:1049. doi: 10.1186/s12889-024-18498-7 (PMC11020674; doi:10.1186/s12889-024-18498-7)
Supplement: Supplementary file 1 — Supplementary Material 1 [file 12889_2024_18498_MOESM1_ESM.docx]

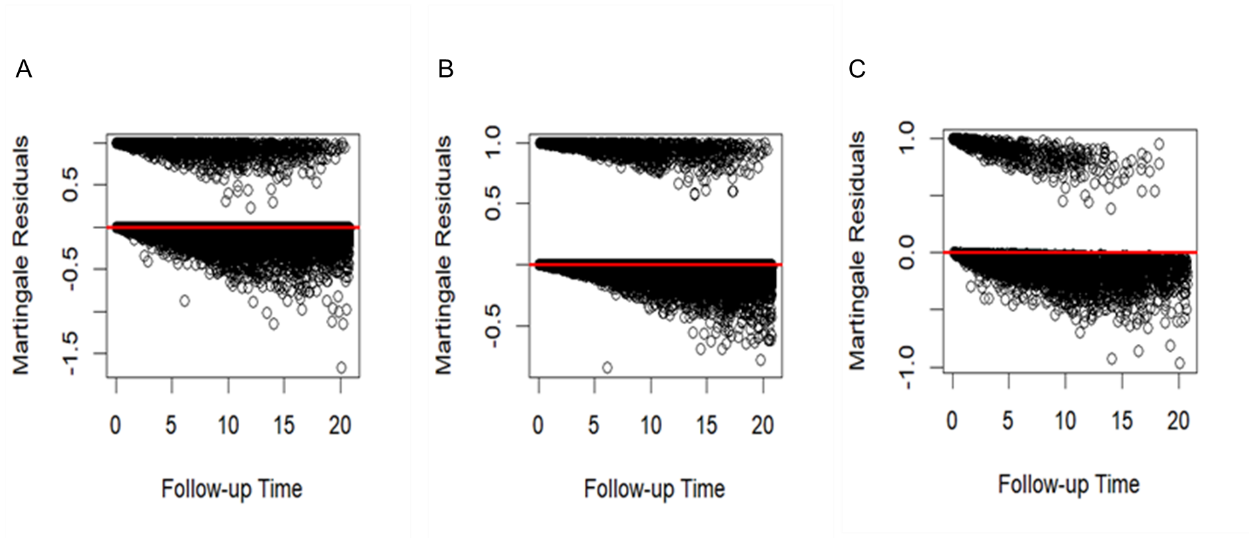


**Supplemental figure:** Martingale residuals plots for survival analysis showed the relationship between follow-up time and Martingale residuals from a proportional hazards model. A: Cox proportional hazards model to explore the association between cardiovascular conditions and cancer mortality among all participants. B: Cox proportional hazards model to explore the association between cardiovascular conditions and cancer mortality among cancer participants. C: Cox proportional hazards model to explore the association between cardiovascular conditions and cancer mortality among non-cancer participants.
